# Diet and Physical Activity as Determinants of Lifestyle Chosen by Women from Southern Poland

**DOI:** 10.3390/ijerph15102088

**Published:** 2018-09-22

**Authors:** Agnieszka Ostachowska-Gasior, Emilia Kolarzyk, Renata Majewska, Anna Gasior, Jacek Kwiatkowski, Izabela Zaleska

**Affiliations:** 1 Department of Hygiene and Dietetics, Jagiellonian University Medical College, 7 Kopernika St., Krakow 31-034, Poland; mykolarz@cyf-kr.edu.pl (E.K.); jackwi@poczta.onet.pl (J.K.); 2 Cracow High School of Health Promotion, 73 Krowoderska St., Krakow 31-158, Poland; 3 Department of Epidemiology, Chair of Epidemiology and Preventive Medicine, Jagiellonian University Medical College, 7A Kopernika St., Krakow 31-034, Poland; rmajewska@cm-uj.krakow.pl; 4 Department of Molecular Biology and Biotechnology, University of Sheffield, Western Bank, Sheffield S10 2TN, UK; ann.gasior@gmail.com; 5 The Faculty of Motor Rehabilitation, University of Physical Education in Cracow, Jana Pawla II 78 Road, Krakow 31-571, Poland; izaleska@icloud.com

**Keywords:** women health, lifestyle, physical activity, quality of diet, questionnaire

## Abstract

The aim of the study was to assess the relationship between the lifestyle of Polish women (characterized by the quality of diets and levels of reported physical activity) and their characteristics such as age, place of residence, physical activity at work or school, reported health status, and BMI. The sample consisted of 882 women from Southern Poland. Diet quality and the level of physical activity were evaluated by the Nutrition Beliefs Questionnaire established by the Polish Academy of Sciences. The lifestyle category (healthy, moderate, or unhealthy) was based on “Prohealthy Diet Index-10” and participant’s self-assessed physical activity during their leisure-time. The lifestyle category was significantly associated with age, BMI, physical activity at work/school, and health. Moderate lifestyle (high or moderate levels of physical activity combined with low prohealthy diet) was the most commonly found classification in examined women. Age (>35 years old) and overweight are the main factors determining unhealthy lifestyle behavior. Healthy lifestyle is more often chosen by the women from big cities. More intensive efforts should be undertaken to increase the knowledge and awareness of the health benefits of a healthy lifestyle. The main goal should be concentrated on increasing the level of physical activity, especially in leisure time, and promoting the tenets of a well-balanced diet.

## 1. Introduction

Unhealthy dietary habits, insufficient physical activity, and sitting for excessively long periods of time are known risk factors for premature mortality mainly caused by coronary heart disease (CHD) and major, noncommunicable diseases [1] such as obesity and diabetes [2,3] as well as some kinds of cancer (e.g., breast cancer [4,5] or colorectal cancer [6]). High CHD mortality has been linked to unhealthy dietary habits in the Former Soviet Union (FSU) and three Central and Eastern European (CEE) populations [7]. In Poland, Multicenter All-Polish Examinations of the WOBASZ II (14,769 persons aged 20–74; average period of observation—8.2 years) and WOBASZ Senior (1,096 persons aged above 74; average period of observation—5 years) indicated that the mortality associated with a CHD was the most frequent cause of death (568 out of overall 1446 death cases; 38% of them were among men and 31% among women) [8]. CHD risk is highly dependent on physical activity and diet. Lack of physical activity is a common problem [9]. Estimations showed that globally, as much as one third of adult population is not sufficiently physically active (17% in South-East Asia and approximately 43% in the Americas and eastern Mediterranean area). It has been shown that the activity level decreases with age, and is higher in men than in woman, which is especially noticeable in high-income countries [10]; however it is not restricted to these countries. Cross-sectional, community-based data obtained from low and middle-income countries by the World Health Survey showed that 29.2% of examined individuals (average age 38.4 years; 50.4% women, 49.6% men) exhibit low physical activity level (<150 min per week) [11]. Population-based studies also provide data allowing for comparison between different countries, which showed high levels of sedentary time and physical inactivity across European adults [12]. In the past year, the British Heart Foundation evaluated levels of physical inactivity and sedentary behavior in adults across the UK (Physical Inactivity Report 2017). The data in this report suggests that large numbers of people in the UK still do not meet recommendations for physical activity, which may lead to increased risk of CHD. In Poland, the results of the NATPOL 2011 survey showed that among the Polish population levels of physical activity are also not satisfactory, and in 2013 the physical activity level of less than a half of a population of working adults exceeded 30 minutes on most days of the week and less than a one third of the examined population chose to commute to work or school by foot or bike [13]. Many researches examining physical activity of Polish adults have been conducted over the years of 2003 to 2014. The general trend was the everyday activity decreased in both men and women from 37.4% to 27.3% and 32.7% to 28.3% in men and women, respectively. None or occasional activity increased from 49.6% to 56.8% in men, and remained stable in women (55.2% vs. 54.9%). No significant changes were observed in the occupational activity of men between the surveys, while the percentage of women with a sedentary work environment increased from 43.4% to 49.4% (*p* < 0.01) [14]. A significant proportion of Poles (5690 subjects examined: 2554 men and 3136 women) had improper dietary habits and their diet was not well-balanced. The intake of fats, saturated and polyunsaturated fatty acids, dietary cholesterol, and fiber was incompatible with recommended doses and the quality of diet of the majority of Polish adults falls far short of the recommendations established for the prevention of CHD [15]. It is important to notice that comparing dietary habits and physical activity levels worldwide is difficult because the methods of assessment of these behaviors are not harmonized and standardized in some countries [16]. A multidisciplinary DEDIPAC KH consortium of researchers from 12 European countries (Austria, Belgium, Finland, France, Germany, Italy, Ireland, Norway, Poland, Spain, The Netherlands, and The United Kingdom) attempted to resolve this problem [17]. In Poland, the standardized principles for assessing diet quality and the level of physical activity at work/school and in a leisure time were established by the Behavioural Nutrition Team Committee of Human Nutrition, Polish Academy of Sciences, which created the Dietary Habits and Nutrition Beliefs Questionnaire and the Manual for Developing of Nutritional Data for nationwide application [18]. According to the Nutrition Beliefs Questionnaire diet quality can be assessed by two indexes: “Prohealthy Diet Index-10” (pHDI-10), and “Non-Healthy Diet Index-14” (nHDI-14). The pHDI-10 concentrates on foods with potentially beneficial effects on health, and the nHDI-14 includes the foods with potentially negative effects on health. The physical activity level is evaluated in three intensity levels: low, moderate, and high.

The aim of this study was to assess the relationship between the lifestyle of Polish women (characterized by the quality of diet and levels of reported physical activity) and their characteristics such as age, place of residence, physical activity at work or school, reported health status, and BMI categories.

## 2. Materials and Methods

### 2.1. Survey Design and Sample

The study involved 882 women (mean age: 33.6 ± 13.9 years, mean height: 166.0 ± 6.0 cm; mean weight: 63.6 ± 11.9 kg) from the south of Poland. Participants did not have a history of major chronic diseases at baseline and did not require any special diets (i.e., severe food allergies or diabetes). The sample consisted of volunteers recruited by a snowball method between 2016 and 2017 [19]. All subjects gave their informed consent for inclusion before they participated in the study, which was conducted in accordance with the Declaration of Helsinki, and The Committee for Human Research of Jagiellonian University, Medical College in Krakow approved the protocol of presented study. This kind of study, in accordance with Polish legal regulations, is not considered a medical experiment and Ethical Committee agreement is not obligatory for medical doctors and dentists (“Dziennik Ustaw” 2011 with modifications; no. 277, line 1634). The diet quality was assessed by the pHDI-10 which focuses on 10 food groups with potentially beneficial effects on health: whole meal bread; buckwheat and oatmeal; milk; fermented milk drinks; fresh cheese curd products; fish; legume-based foods; fruit; vegetables; and white-meat-based dishes [18]. The following categories regarding the frequency of consumption of previously mentioned food groups and corresponding indicators expressed as times per day were established: never—0; 1–3 times a month—0.06; once a week—0.14; few times a week—0.5; once a day—1; few times a day—2. The pHDI-10 was equal to the sum of the frequency of 10 food groups consumption (range 0–20). The intensity of the prohealthy diet was evaluated in three intervals: low intensity prohealthy diet (0–6.66 points), moderate intensity prohealthy diet (6.67–13.33 points), and high intensity prohealthy diet (13.34–20.0 points). The level of declared physical activity at work or at school was calculated according to the following criteria. Low: less than 30% of time is active; moderate: 50% of time is sedentary and 50% is active; high: more than 70% of day is active or physical labor of high intensity. In our analyses we considered women exhibiting moderate and high levels of activity as active (at work or at school). The criteria for assessment of physical activity during leisure time were: low (mostly sedentary, watching TV, reading newspapers/books, light household duties, walking for 1–2 h/week); moderate (walking, cycling, workout, gardening or other light physical activities for 2–3 h/week); high (cycling, running, gardening and other sport/recreational activities that require physical activity for longer than 3 h/week) [19]. Criteria for women’s characteristics: age according to human development stages: <35 years old as young adulthood and ≥35 years old as middle-age and elderly; place of residence: village, small town (up to 100,000 inhabitants), big city (over 100,000 inhabitants); self-assessed health status in comparison to other people their age: worse than others; the same as others; better than others; Body Mass Index (BMI) categories according to WHO categories: <18.5 kg/m^2^—underweight, 18.5–24.99 kg/m^2^—normal weight, 25.0–29.9 kg/m^2^—overweight, ≥30 kg/m^2^—obesity, physical activity at work/school: inactive—low level of reported physical activity, active—high or moderate level of declared physical activity.

### 2.2. Lifestyle Definition

Lifestyle category was based on pHDI-10 values and participant’s self-assessed physical activity level during their leisure time.

As only three women were categorized as “physically inactive with a prohealthy diet” they were excluded from further considerations. The final analyses were based on three categories of lifestyle:

- Healthy lifestyle (high or moderate levels of declared physical activity and moderate and high prohealthy diet);
- Moderate lifestyle (high or moderate levels of declared physical activity and low prohealthy diet);
- Unhealthy lifestyle (low levels of declared physical activity and low prohealthy diet).

### 2.3. Statistical Analysis

All characteristics were presented as frequencies with a percentage distribution. Associations between lifestyle category and characteristics such as age, residential place, physical activity at work or school, BMI categories, and self-assessed health status were verified using a χ^2^ test. To assess if women’s lifestyle choices could be independently influenced by the above-mentioned factors a multinomial logistic regression was used. First, simple multinomial logistic regression models with only one independent factor were presented. Then, they were followed by one multiple multinomial logistic regression model with all the studied factors. Predicted probabilities (with 95% confidence intervals) of choosing a healthy, moderate, and unhealthy lifestyle were based on multiple multinomial logistic models which were calculated for each category of self-assessed health status, place of residence, and physical activity at work/school, holding all other variables at their means.

All tests were two-tailed, and significance was set to *p* < 0.05. Analyses were performed using Stata/IC 13.1 (StataCorp LP., College Station, TX, USA).

## 3. Results

The study group consisted of 882 women, where 32.0% were physically active with prohealthy diet (healthy lifestyle), 29.5% were inactive with low prohealthy diet (unhealthy lifestyle), 38.2% were active with low prohealthy diet (moderate lifestyle), and only 0.3% (*n* = 3) were inactive with prohealthy diet. As the last group included only three women, it was excluded from further analyses. Table 1 presents characteristics of all analysed study samples (*n* = 879) including lifestyle category.

Lifestyle category was significantly associated with age, BMI, physical activity at work or at school, and declared health status. Women over 35 years old were more likely to exhibit “unhealthy” lifestyle, than younger women, who more often exhibited “moderate” lifestyle (OR = 0.56; 95% CI: 0.40–0.78). Overweight and obese women were more likely to exhibit “unhealthy” lifestyle than women with normal weight (OR = 0.46; 95% CI: 0.32–0.66). Women who self-assessed their health as worse compared to the health status of their peers were also more likely to exhibit “unhealthy” lifestyle (OR = 0.31; 95% CI: 0.20–0.48). In contrast, underweight women active at work or school were more likely to exhibit “moderate” lifestyle, than women active at work or school with normal weight, who were more likely to exhibit “unhealthy” lifestyle (OR = 1.74; 95% CI: 1.25–2.42 and OR = 2.62; 95% CI: 1.18–5.84, respectively). It was more likely that a woman exhibited “healthy” rather than “unhealthy” lifestyle if she lived in a big city than in a village (OR = 1.61; 95% CI: 1.05–2.47), was underweight (OR = 1.61; 95% CI: 1.60–7.79), was active at work or school (OR = 4.56; 95% CI: 3.11–6.69), and self-assessed her health as better compared to her peers (OR = 2.44; 95% CI: 1.49–4.01). Women who self-assessed their health as worse than their peers were less likely to choose “healthy” rather than “unhealthy” lifestyle (OR = 0.31; 95% CI: 0.18–0.48) (Table 2; simple models). Underweight and being active at work were the factors that independently determined choice of “moderate” rather than “unhealthy” lifestyle (OR = 2.63; 95% CI: 1.15–6.01 and OR = 1.91; 95% CI: 1.34–2.72, respectively), while obese or overweight women and those who assessed their health status as worse than their peers were less likely to exhibit moderate rather than unhealthy lifestyle (OR = 0.62; 95% CI: 0.40–0.97 and OR = 0.37; 95% CI: 0.23–0.59, respectively) (Table 2; multiple model).

**Figure 1:**
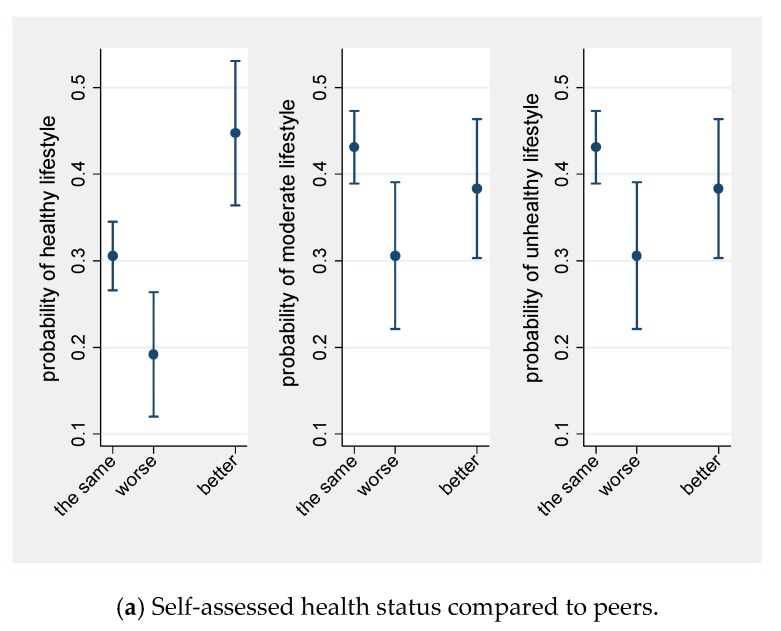

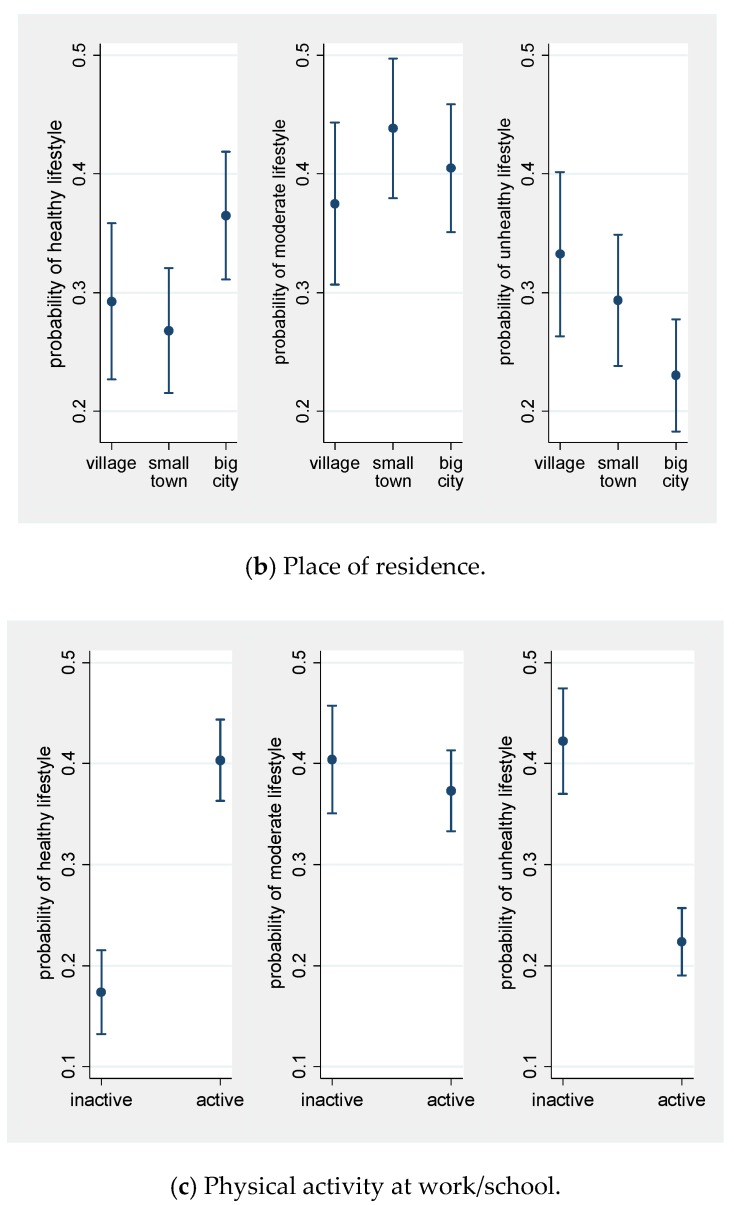
Predicted probabilities (with 95% confidence intervals) of healthy, moderate, and unhealthy lifestyle at each category of (**a**) health status, (**b**) place of residence, and (**c**) activity at work/school.

Probability of moderate and healthy lifestyle category against health status, place of residence, and activity at work/school are presented at Figure 1.

## 4. Discussion

Lifestyle-related factors are connected not only with chronic disease development but also with premature death [20]. Unfortunately, all these factors are usually considered individually, and therefore examinations assessing the combined effect of the lifestyle behaviors on morbidity and mortality should be developed. This research attempted to analyze the influence of lifestyle-related factors, physical activity, and diet quality on the self-assessed health status of examined Polish women.

The meta-analysis conducted by Loef and Walach, which included over 500,000 individuals from 18 cohorts, showed that relative risk of mortality decreases proportionally to a higher number of analyzed healthy lifestyle factors [21]. The following lifestyle risk factors are most frequently analyzed: inadequate diet, insufficient physical activity, tobacco use, and excessive alcohol use. Additionally, inadequate diet and insufficient physical activity usually leads to obesity, which is now considered to be a major risk factor for most noncommunicable diseases [22]. In 2017, the WHO reported that worldwide obesity has increased by 300% since 1975, and in 2016, almost 2 billion (39%) adults (18 years and older) were overweight, of which 650 million (13%) suffered from obesity. The WHO emphasized the fact that differences in lifestyle between men and women caused by social and sex-specific biological factors should also be taken into consideration [1]. Results of our current research confirmed our previous results which showed that physical activity at work does not interfere with leisure time activity [23]. Moreover, women working in a sedentary environment do not spend their free time exercising. Additionally, activity at work or school turned out to be a significantly important factor in choosing healthy lifestyle. Biernat and Piatkowska suggested that leisure-time physical activity, for many occupations, is the only way to overcome sedentary behavior patterns [24], however The Polish Public Opinion Research Center survey showed that physical activity is an unpopular choice of free-time activity among Polish nationals [25]. The literature indicates that leisure-time physical activities undertaken with the duration and frequency recommended for maintaining health might, regardless of the intensity of occupational physical activity, raise individual capacity and physical fitness [26]. A dose-response relationship has been observed between being active, even to a modest level, and being inactive or exhibiting sedentary lifestyle. The greatest health benefits are usually observed in the previously sedentary individuals deciding to change their lifestyle into a more active one [27]. The quality of diet assessed in previously published results (obtained from the same set of participants who were examined in this research) in most cases did not fulfill the prohealthy criteria enclosed in the pHDI-10 [18]. The consumption of healthy food products in all subgroups of women—underweight, overweight/obese, and women with normal body weight—was of insufficient level in comparison to current Polish nutritional recommendations [28]. In the current study, diet was only one part of the lifestyle determination process. The second part was composed of the levels of physical activity in leisure time. Healthy lifestyle was defined by joint effects of prohealthy diet and prohealthy levels of physical activity in leisure time. Our study showed that women with high levels of physical activity do not always maintain a healthy diet, whereas women exhibiting healthy dietary habits are more often physically active. It can then be concluded that leisure-time physical activity does not increase the probability of maintaining a healthy diet. Only three women having a prohealthy diet were physically inactive. Opposite to this finding, 337 women reporting high or moderate levels of physical activity applied a diet with a low intensity of prohealthy characteristics. A direct effect of diet on physical activity was also confirmed in another examination where it was postulated that individual weight-related factors, such as eating habits, are chief motivators for engaging in physical activity [29]. In our examination a healthy lifestyle was more frequently observed among women living in big cities in comparison to women living in rural areas, which is consistent with previous research. The Spanish National Health Survey, focusing on women >16 years old, reported that women from medium-sized urban or rural areas less frequently used the preventive public health care services, and their lifestyle behaviors, such as leisure time physical activity, smoking, alcohol use, or dietary pattern were significantly less beneficial for their health than for the women living in metropolitan areas [30]. The examination of 1818 United States adults reported that suburban, higher income residents were more likely to follow recommendations for proper level of physical activity than rural, lower income residents [31]. Barriers to leisure time physical activity experienced by people living in rural areas include low availability of sport facilities and long distance to urban facilities, lower economic status, or inconvenient working hours [32]. Physical activity may also be affected by social inequalities. The prevalence of leisure-time physical inactivity among the beneficiaries of the welfare group was 1.5 times higher than in the general Polish population [33]. Biernat and Buchholtz’s examination, which included 1260 low-physically active adult Poles (age between 15 and 69 years), showed that lack of physical activity was especially prevalent in individuals living in rural areas or town dwellers, and was often associated with poor educational level and restrained labor market opportunities [34]. Later results of these authors obtained from LTPA (Leisure-Time Physical Activity) survey in Poland, commissioned by the Polish Ministry of Sport and Tourism (2014–2015) indicated that the World Health Organization prohealth leisure-time physical activity recommendations were not followed by 66% of farmers and 49% of other dwellers in rural areas. The amount of physical activity was in general negatively correlated with age, however interestingly, a higher level of activity has been observed for the >60 years old group, than for people aged 50–59 [35]. Our results showed that underweight women were more likely to lead a healthy lifestyle than those with normal weight, which was unexpected. However, this observation might not be accurate, due to the small number of women (*n* = 72) with BMI <18.5 kg/m^2^ taking part in our examination. Additionally, research demonstrated that women, especially young women, are not satisfied with their weight and body, and often overestimated their weight, and therefore aim to have a lower BMI [36]. Even those with a BMI indicating ‘underweight’ desire to improve their image and make a decision about increasing their level of physical activity. The Longitudinal Study of Adult Health (ELSA-Brasil) showed that women’s dissatisfaction with their bodies was mediated by insufficient physical activity and unhealthy dietary habits, however, only women with insufficient physical activity levels considered unhealthy eating habits as the reason for their body dissatisfaction. Lack of satisfaction with body image was often observed in women who self-assessed their health as poor [37]. In our examination women who assessed their health as being worse than their peers more frequently chose unhealthy than healthy lifestyle. The results of our examination also indicated that overweight or obesity were not independent factors determining lifestyle category. Estimation of nutritional status using the Body Mass Index is one of the limitations of our study. BMI does provide accurate information about weight–height proportions; however, objective measurements of body mass components (fat and lean body mass and total body water) should be performed for reliable estimation of particular category of nutritional status end especially for estimation of overweight etiology. In the case of a physically active group, overweight may not be caused by excess fat tissue, but it may be induced by muscle mass. In our examination the measurement of body composition was not possible. We are also fully aware that results were obtained from declarations and self-assessment instead of objective measurements One of the merits of this study was fact that the examination was performed using a standardized questionnaire created by the Behavioural Nutrition Team Committee of Human Nutrition, the Polish Academy of Sciences, which is recommended for all Polish researchers in order to allow for comparison of results obtained by different authors.

## 5. Conclusions

1. Two opposite models of lifestyle—prohealthy and unhealthy are equally frequent among the examined group of women from southern Poland.
2. Moderate lifestyle (high or moderate levels of physical activity combined with low prohealthy diet) was the most commonly found in the examined group of women.
3. Age (>35 years old) and overweight are the main factors determining unhealthy lifestyle behavior.
4. Women who self-assessed their health status as worse than their peers were not motivated to increase their level of physical activity or to improve their dietary habits.
5. Healthy dietary habits are related to the type of leisure-time activity (women with prohealthy diet more frequently are physically active during their leisure time), and moderate and high physical activity levels do not increase the probability of making dietary choices beneficial for health.
6. Healthy lifestyle is more often chosen by women from big cities in comparison to those from rural areas.
7. More intensive efforts should be undertaken to increase the knowledge and awareness of health benefits of a healthy lifestyle. Special population-wide intervention programs and new strategies should be implemented. These activities should be addressed mainly for rural women, and the main goal should be concentrated on increasing the level of physical activity, especially in leisure time and promoting the rules of a proper, well-balanced diet.

## Figures and Tables

**Table 1:** Characteristics of study sample including lifestyle categories.

			Lifestyle						*p*
	Total *N* = 879		Healthy *N* = 282		Moderate *N* = 337		Unhealthy *N* = 260		
Age									
<35 years	521	59.3%	164	58.2%	222	65.9%	135	51.9%	0.002
≥35 years	358	40.7%	118	41.8%	115	34.1%	125	48.1%	
Residential place									
Village	214	24.3%	63	22.3%	79	23.4%	72	27.7%	0.126
Small town (up to 100,000 inhabitants)	306	34.8%	88	31.2%	123	36.5%	95	36.5%	
Big city (over 100,000 inhabitants)	359	40.8%	131	46.5%	135	40.1%	93	35.8%	
BMI									
Underweight	72	8.2%	32	11.3%	32	9.5%	8	3.1%	<0.001
Normal weight	569	64.7%	175	62.1%	238	70.6%	156	60.0%	
Overweight	167	19.0%	56	19.9%	52	15.4%	59	22.7%	
Obesity	71	8.1%	19	6.7%	15	4.5%	37	14.2%	
Physical activity at work/school									
Inactive	318	36.2%	54	19.1%	129	38.3%	135	51.9%	<0.001
Active	561	63.8%	228	80.9%	208	61.7%	125	48.1%	
Self-assessed health status									
Worse than their peers	134	15.2%	25	8.9%	35	10.4%	74	28.5%	<0.001
The same as their peers	590	67.1%	184	65.2%	246	73.0%	160	61.5%	
Better than their peers	155	17.6%	73	25.9%	56	16.6%	26	10.0%	

**Table 2:** Chance of a healthy and moderate lifestyle category against different factors.

	Simple Models				Multiple Model			
	Healthy vs. Unhealthy		Moderate vs. Unhealthy		Healthy vs. Unhealthy		Moderate vs. Unhealthy	
Characteristics	OR	95% CI	OR	95% CI	OR	95% CI	OR	95% CI
Age ≥35	0.78	0.55–1.09	**0.56**	**0.40–0.78**	0.72	0.46–1.12	0.67	0.44–1.01
Residence place								
Small town vs. village	1.06	0.68–1.65	1.18	0.78–1.79	1.04	0.64–1.69	1.32	0.85–2.06
Big city vs. village	**1.61**	**1.05–2.47**	1.32	0.87–2.00	**1.80**	**1.12–2.90**	1.56	1.00–2.43
Active at work/school	**4.56**	**3.11–6.69**	**1.74**	**1.25–2.42**	**4.95**	**3.29–7.45**	**1.91**	**1.34–2.72**
BMI								
Underweight vs. normal weight	**3.57**	**1.60–7.79**	**2.62**	**1.18–5.84**	**3.37**	**1.43–7.94**	**2.63**	**1.15–6.01**
Overweight or obesity vs. normal weight	0.70	0.48–1.01	**0.46**	**0.32–0.66**	0.91	0.57–1.45	0.62	0.40–0.97
Self-assessed health status								
Worse vs. the same	**0.29**	**0.18–0.48**	**0.31**	**0.20–0.48**	**0.33**	**0.19–0.56**	**0.37**	**0.23–0.59**
Better vs. the same	**2.44**	**1.49–4.01**	1.41	0.84–2.32	**2.28**	**1.34–3.86**	1.38	0.82–2.33

OR—odds ratio; 95% CI—95% confidence interval; statistically significant results were bolded
